# Promise and Challenge of DNA Barcoding in Venus Slipper (*Paphiopedilum*)

**DOI:** 10.1371/journal.pone.0146880

**Published:** 2016-01-11

**Authors:** Yan-Yan Guo, Lai-Qiang Huang, Zhong-Jian Liu, Xiao-Quan Wang

**Affiliations:** 1 Shenzhen Key Laboratory for Orchid Conservation and Utilization, The National Orchid Conservation Center of China and The Orchid Conservation and Research Center of Shenzhen, Shenzhen, China; 2 Center for Biotechnology and BioMedicine, Graduate School at Shenzhen, Tsinghua University, Shenzhen, China; 3 State Key Laboratory of Systematic and Evolutionary Botany, Institute of Botany, Chinese Academy of Sciences, Beijing, China; Chinese Academy of Medical Sciences, Peking Union Medical College, CHINA

## Abstract

Orchidaceae are one of the largest families of flowering plants, with over 27,000 species described and all orchids are listed in CITES. Moreover, the seedlings of orchid species from the same genus are similar. The objective of DNA barcoding is rapid, accurate, and automated species identification, which may be used to identify illegally traded endangered species from vegetative specimens of *Paphiopedilum* (Venus slipper), a flagship group for plant conservation with high ornamental and commercial values. Here, we selected eight chloroplast barcodes and nrITS to evaluate their suitability in Venus slippers. The results indicate that all tested barcodes had no barcoding gap and the core plant barcodes showed low resolution for the identification of Venus slippers (18.86%). Of the single-locus barcodes, nrITS is the most efficient for the species identification of the genus (52.27%), whereas *mat*K + *atp*F-*atp*H is the most efficient multi-locus combination (28.97%). Therefore, we recommend the combination of *mat*K + *atp*F-*atp*H + ITS as a barcode for Venus slippers. Furthermore, there is an upper limit of resolution of the candidate barcodes, and only half of the taxa with multiple samples were identified successfully. The low efficiency of these candidate barcodes in Venus slippers may be caused by relatively recent speciation, the upper limit of the barcodes, and/or the sampling density. Although the discriminatory power is relatively low, DNA barcoding may be a promising tool to identify species involved in illegal trade, which has broad applications and is valuable for orchid conservation.

## Introduction

DNA barcoding uses short DNA sequences to identify species [1,2]. Barcoding is a practical, simple, and quick method compared to traditional methods, but there are pros and cons for DNA barcoding [3–10]. Because of its potential application in several areas of biology, such as species identification, biodiversity assessment, plant conservation, trade control to biomedicine, forensics, and many other applications, DNA barcoding has undergone significant development and growth and hundreds of articles have been published. Therefore, many biologists and other end users have positive attitudes towards DNA barcoding.

Because of the frequent structural variation, low mutation rate, and horizontal gene transfer of plant mitochondrial genome [11,12], greater attention was paid to the chloroplast DNA barcodes in plants. A series of chloroplast fragments have been recommended as barcodes, such as the coding regions, *acc*D, *mat*K, *ndh*J, *rbc*L, *rpo*C1, *rpo*B, and *ycf*5, and noncoding regions, *atp*F-*atp*H, *psb*K-*psb*I, *trn*H-*psb*A, and the *trn*L intron. Because plant chloroplast genes have a lower mutation rate than animal mitochondrial genes, a multi-locus approach is generally adopted for plant barcodes [2,13–19]. For example, Kress *et al*. [2] proposed that the commonly used ITS spacer and the highly variable *trn*H-*psb*A region be used in combination to identify flowering plants. Chase *et al*. [15] outlined two three-region options, *rpo*C1 + *rpo*B + *mat*K and *rpo*C1 + *mat*K + *trn*H-*psb*A. Finally, the CBOL Plant Working Group [18] recommended the combination of *rbc*L and *mat*K as a core plant barcode.

Although there is considerable debate regarding DNA barcoding, the technique remains under active development and many plant groups have been tested, such as *Aspalathus* (Fabaceae) [20], *Crocus* and *Sisyrinchium* (Iridaceae) [21,22], *Carex* (Cyperaceae) [23], *Tolpis* (Asteraceae) [24], *Picea* (Pinaceae) [25], *Alnus* (Betulaceae) [26], Lemnaceae [27], *Panax* (Araliaceae) [28], *Ligustrum* (Oleaceae) [29], ferns [30], *Prunus* (Rosaceae) [31], *Gaultheria* (Ericaceae) [32], Juglandaceae [33], *Pedicularis* (Orobanchaceae) [34], Bromeliaceae [35], *Parnassia* (Parnassiaceae) [36], *Lysimachia* (Myrsinaceae) [37], *Gossypium* (Malvaceae) [38], *Thymus* (Lamiaceae) [39], *Populus* (Salicaceae) [40], Podocarpaceae [41], and *Angelica* (Umbelliferae) [42]. The resolution varied greatly among different plant lineages; for example, the resolution of single- and multi-locus was only 60% in *Carex* [23]. However, in other groups, the selected loci showed high resolution and the combination of ITS + *trn*H-*psb*A can discriminate 90.0% species of *Parnassia* [36], whereas ITS2 can resolve 98.93% of cotton species [38].

Orchidaceae are one of the largest families of flowering plants and all orchids are listed in CITES. However, to date the barcoding of orchids is rather limited in number and scope [43–51]. Lahaye *et al*. [44] proposed *mat*K as barcode for the identification of the flowering plants based on data from >1,000 species of Mesoamerican and South Africa orchids. In addition, the species in some genera have been sparsely sampled, for example, Yao *et al*. [47] studied 17 species of *Dendrobium*, while Parveen *et al*. [48] sampled only eight species of *Paphiopedilum*. Because these studies were based on relatively sparse sampling, the question remains: when more samples are added to these large, diverse genera, will the resolution remain high? Orchid DNA barcoding is far from resolved and more samples and genera should be tested and *Paphiopedilum* provides an opportunity to explore these questions.

*Paphiopedilum* Pfitzer (Venus slipper) is the largest genus of slipper orchids, with 96 accepted species (data collected from KBG, 01/2014) and is an ideal group to evaluate the suitability of candidate barcodes for the conservation of plants. Almost all species of the genus have showy flowers and long flowering periods, often up to several months and have been cultivated widely since the 19^th^ Century [52,53]. However, the ornamental and commercial value of the genus has caused over-collection and illegal poaching and trade [54,55]. For example, *Paphiopedilum lawrenceanum* has 120 years of cultivation history, but there are no wild populations because of over-collection [56]. *Paphiopedilum vietnamense* was only discovered in 1997 and is critically endangered in nature [57,58] and all of the species distributed in Vietnam are disappearing rapidly [54]. In addition, the customs and quarantine inspectors often cannot differentiate between rare and common species when not in flower [52]. The young seedlings of *Paphiopedilum* are very similar and are difficult to differentiate. Thus, morphological assessments are time-consuming, expensive, and require skilled labor [59]. Therefore, DNA barcoding might be used to solve these problems.

In this study, our objectives are as follows: 1) test the performance of the core plant barcode in Venus slippers; 2) evaluate the discriminatory power of nine single-loci (*acc*D, *mat*K, *rbc*L, *rpo*C2, *ycf*1, *atp*F-*atp*H, *atp*I-*atp*H, ITS) and multi-locus combinations with dense taxon sampling and test whether an upper limit exists in the barcodes; and 3) discuss the factors that affect barcoding success.

## Materials and Methods

### Plant sampling

We used the data in Guo *et al*. [60] for our analysis with two unknown samples excluded. A total of 107 samples representing 77 *Paphiopedilum* species were used to test the species resolution, 22 of which were represented by two or more individuals and varieties were treated as samples within the same species. These data were supplemented with additional data from GenBank (http://www.ncbi.nlm.nih.gov/genbank/) (S1 Table) to test the upper limit of the barcodes. In total, 359 ITS sequences, 116 *mat*K sequences, 60 *ycf*1 sequences, and 44 *rbc*L sequences were downloaded from GenBank.

### Data analysis

The sequences were aligned with BioEdit [61] and refined manually. First, we analyzed the data from Guo *et al*. [60]. We evaluated the resolution of eight single-locus DNA regions (*acc*D, *mat*K, *rbc*L, *rpo*C2, *ycf*1, *atp*F-*atp*H, *atp*I-*atp*H), six selected two-locus combinations (*rbc*L + *acc*D, *rbc*L + *mat*K, *ycf*1 + *rpo*C2, *ycf*1 + *atp*F-*atp*H, *rpo*C2 + *atp*F-*atp*H, *mat*K + *atp*F-*atp*H), two three-locus combinations (*rbc*L + *mat*K + *atp*F-*atp*H, *trn*S-*trn*fM + *atp*I-*atp*H + *atp*F-*atp*H), and the combined eight cpDNA regions. Then, we evaluated the resolution of ITS and three cpDNA sequence regions (*mat*K, *rbc*L, *ycf*1) with the data downloaded from GenBank. The analysis was performed with the SpeciesIdentifier 1.7.7 program from the TaxonDNA software package [62]. The inter- and intra-specific genetic divergences were calculated following Meyer and Paulay [63] and were used to determine whether a barcoding gap exists. The best match/best close match was used to assess the correct identification of the species [62]. To assess the haplotype accumulation in different datasets, we calculated the accumulation curves for haplotypes in the cpDNA and ITS of *Paphiopedilum* with the SPIDER package in R [64]. Neighbor-joining analysis of the eight combined cpDNAs was performed in MEGA6 [65], with the Kimura-2-parameter distance option and 1000 replicates.

## Results

**Fig 1:**
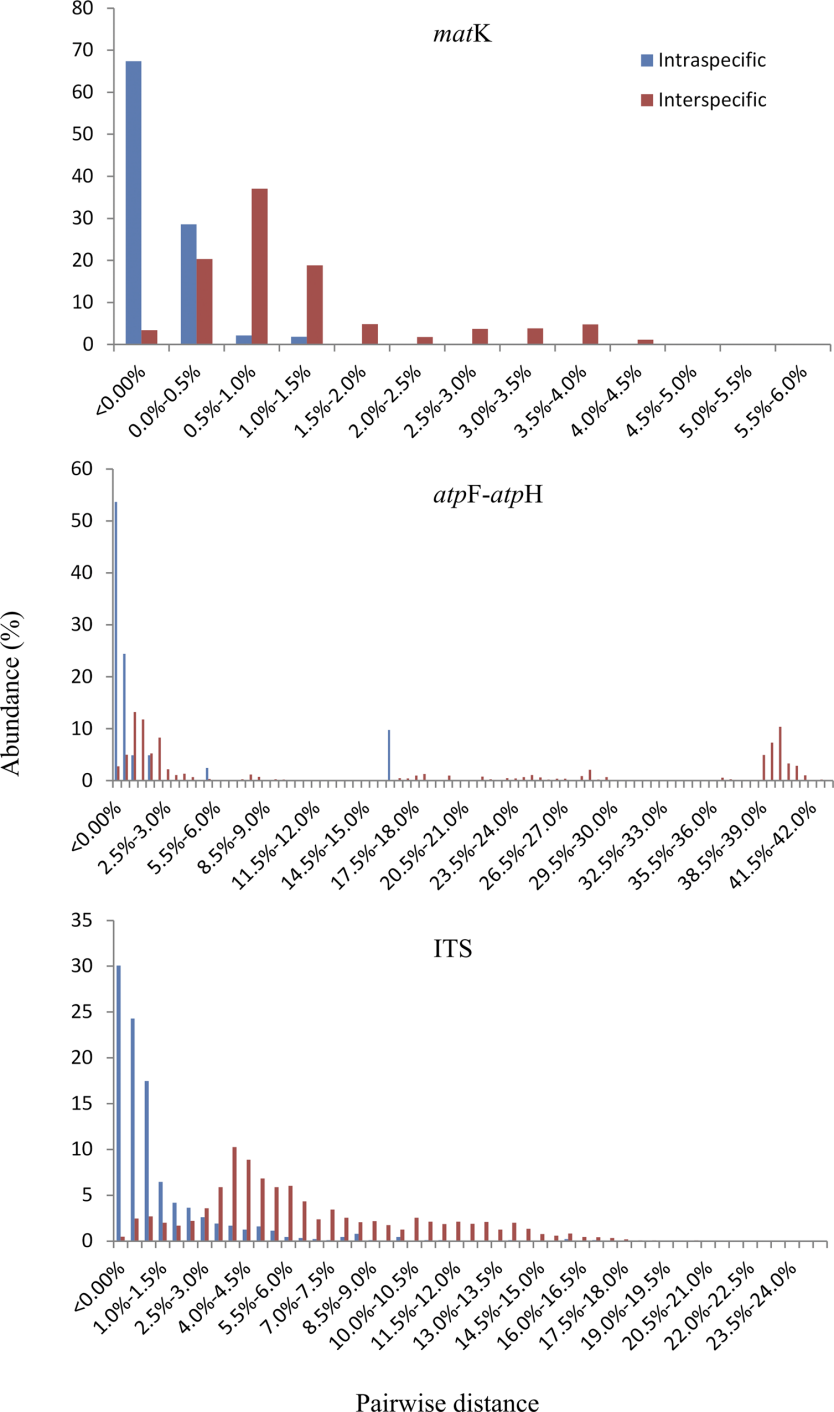
Distribution of the relative abundance of intra- and interspecific K2P for the candidate barcode marker.

The number of sequences analyzed and the sequence lengths are listed in Table 1. The attendant datasets included approximately 70–90% of the accepted species of Venus slipper. The species were best represented by the ITS dataset (72/85), followed by *mat*K (55/84), and *ycf*1 (52/79), but other datasets have lower intra-species sampling. The intra- and interspecific distance ranges overlapped and all tested barcodes had no barcoding gap (Fig 1). The summary of the single- and multi-locus barcode resolution is listed in Table 2. The ITS has the highest discriminatory power of the single-locus barcodes (52.27%) and approximately half the attendant sequences were identified successfully. In the single-locus analysis of the five coding cpDNA regions, *rpo*C2 has the highest resolution (25.74%), followed by *ycf*1, *mat*K, and *acc*D (22.42%, 15.88%, and 14.01%, respectively), whereas *rbc*L has the lowest discrimination rate (3.77%). Of the three intergenic regions, *atp*F-*atp*H has the highest resolution (22.42%), followed by *atp*I-*atp*H, and *trn*S-*trn*fM (19.62% and 13.33%, respectively). Of the multi-locus combinations, except the two two-locus combinations, those with *rbc*L have relatively lower resolutions (14.14% and 18.86%) and the discriminatory power of the other combinations is similar, ranging from 25.74% to 29.52%. The resolution did not increase significantly with the addition of sequence length.

**Table 1:** Sequence information of the genes used in the study.

Data sets	N of sequences/ N of species	Species represented by multiple individuals	Sequence length (bp)	Alignment length (bp)
*acc*D	107/77	22	669–699	723
*mat*K	107/77	22	600–609	619
*mat*K_1	223/84	55	591–609	609
*rbc*L	106/76	22	485	485
*rbc*L_1	147/77	27	485	485
*ycf*1	107/77	22	1525–1777	2041
*ycf*1_1	167/79	52	1525–1777	2047
*rpo*C2	101/73	21	2721–2736	2781
*trn*S-*trn*fM	105/75	22	701–785	903
*atp*I-*atp*H	107/77	22	467–624	914
*atp*F-*atp*H	107/77	22	180–423	577
ITS	352/85	72	588–689	739

**Table 2:** Identification success of analyzed barcodes using SpeciesIdentifier 1.7.7 program under ‘best match’ and ‘best close match’ methods (Meier et al. 2006).

Barcode	No. Sequences	Best match (%)			Best close match (%)				Threshold (%)
		Correct	Ambiguous	Incorrect	Correct	Ambiguous	Incorrect	No match	
*rbc*L (A)	106	4 (3.77)	96 (90.56)	6 (5.66)	4 (3.77)	96 (90.56)	6 (5.66)	0 (0.00)	0.41
*rbc*L_1	150	10 (6.66)	132 (88.00)	8 (5.33)	10 (6.66)	132 (88.00)	8 (5.33)	10 (6.66)	0.4
*acc*D (B)	107 (107)	15 (14.01)	81 (75.70)	11 (10.28)	15 (14.95)	80 (74.76)	10 (9.34)	2 (1.86)	0.41
*mat*K (C)	107 (107)	17 (15.88)	74 (69.15)	16 (14.95)	17 (15.88)	72 (67.28)	13 (12.14)	5 (4.67)	0.65
*mat*K_1	223	73 (32.73)	134 (60.08)	16 (7.17)	73 (32.73)	133 (59.64)	14 (6.27)	3 (1.34)	0.49
*ycf*1 (D)	107 (107)	24 (22.42)	50 (46.72)	33 (30.84)	24 (22.42)	44 (41.12)	20 (18.69)	19 (17.75)	0.14
*ycf*1_1	167	52 (31.13)	64 (38.32)	51 (30.53)	52 (31.13)	64 (38.32)	50 (29.94)	1 (0.59)	5.59
*rpo*C2 (E)	101 (107)	26 (25.74)	42 (41.58)	33(32.67)	26 (25.74)	42 (41.58)	23 (22.77)	10 (9.90)	0.17
*trn*S-*trn*fM (F)	105 (107)	14 (13.33)	85 (80.95)	6 (5.71)	12 (11.42)	71 (67.61)	6 (5.71)	16 (15.23)	0.21
*atp*I-*atp*H (G)	107 (107)	21 (19.62)	61 (57.00)	25 (23.36)	21 (19.62)	61 (57.00)	24 (22.42)	1 (0.93)	14.65
*atp*F-*atp*H (H)	107 (107)	24 (22.42)	62 (57.94)	21 (19.62)	24 (22.42)	62 (57.94)	21 (19.62)	0 (0.00)	16.45
ITS	352	184 (52.27)	113 (32.1)	55 (15.62)	183 (51.98)	112 (31.81)	54 (15.34)	3 (0.85)	4.86
AB	106 (107)	15 (14.14)	76 (71.69)	15 (14.14)	13 (12.26)	59 (55.66)	10 (9.43)	24 (22.64)	0
AC	106 (107)	20 (18.86)	62 (58.48)	24 (22.64)	20 (18.86)	61 (57.54)	24 (22.64)	1 (0.94)	0.54
DE	101 (107)	28 (27.72)	35 (34.65)	38 (37.62)	27 (26.73)	34 (33.66)	28 (27.72)	12 (11.88)	0.21
DH	107 (107)	29 (27.1)	35 (32.71)	43 (40.18)	29 (27.1)	35 (32.71)	42 (39.25)	1 (0.93)	3.74
EH	101 (107)	29 (28.71)	44 (43.56)	28 (27.72)	29 (28.71)	44 (43.56)	28 (27.72)	0 (0.00)	2.84
CH	107 (107)	31 (28.97)	45 (42.05)	31 (28.97)	31 (28.97)	44 (42.05)	32 (28.97)	0 (0.00)	8.26
ACH	106 (107)	30 (28.3)	43 (40.56)	33 (31.13)	30 (28.3)	43 (40.56)	33 (31.13)	0 (0.00)	5.86
ABCDE	101 (107)	26 (25.74)	32 (31.68)	43(42.57)	26 (25.74)	29 (28.71)	31 (30.69)	15 (14.85)	0.2
FGH	105 (107)	31 (29.52)	41 (39.04)	33 (31.42)	31 (29.52)	41 (39.04)	32 (30.47)	1 (0.95)	4.13
ABCDEFGH	100 (107)	29 (28.99)	26 (26.00)	45 (45.00)	29 (28.99)	26 (26.00)	41 (41.00)	4(4.00)	1.21

**Fig 2:**
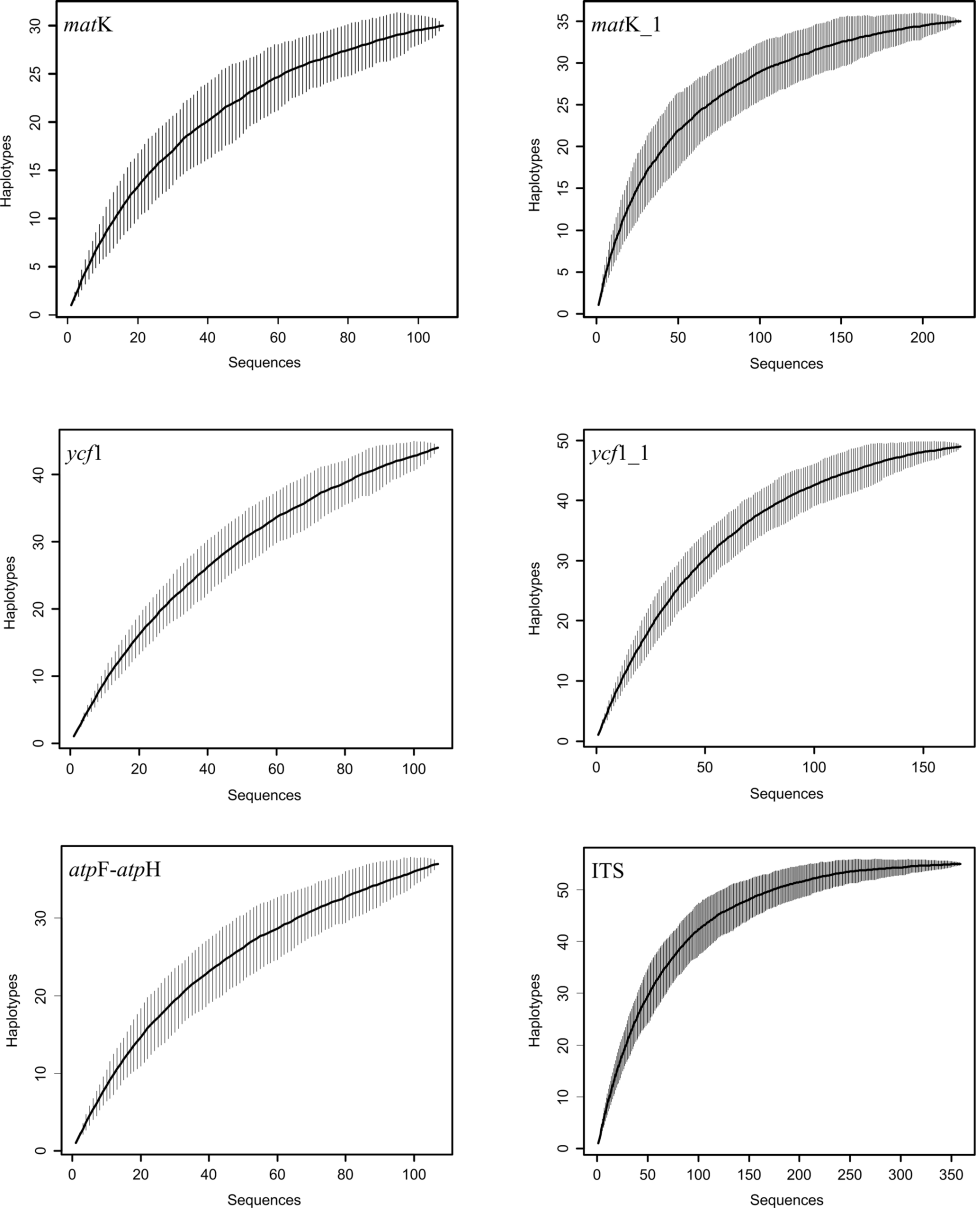
Accumulation curves for haplotypes in cpDNA and ITS in *Paphiopedilum*.

**Fig 3:**
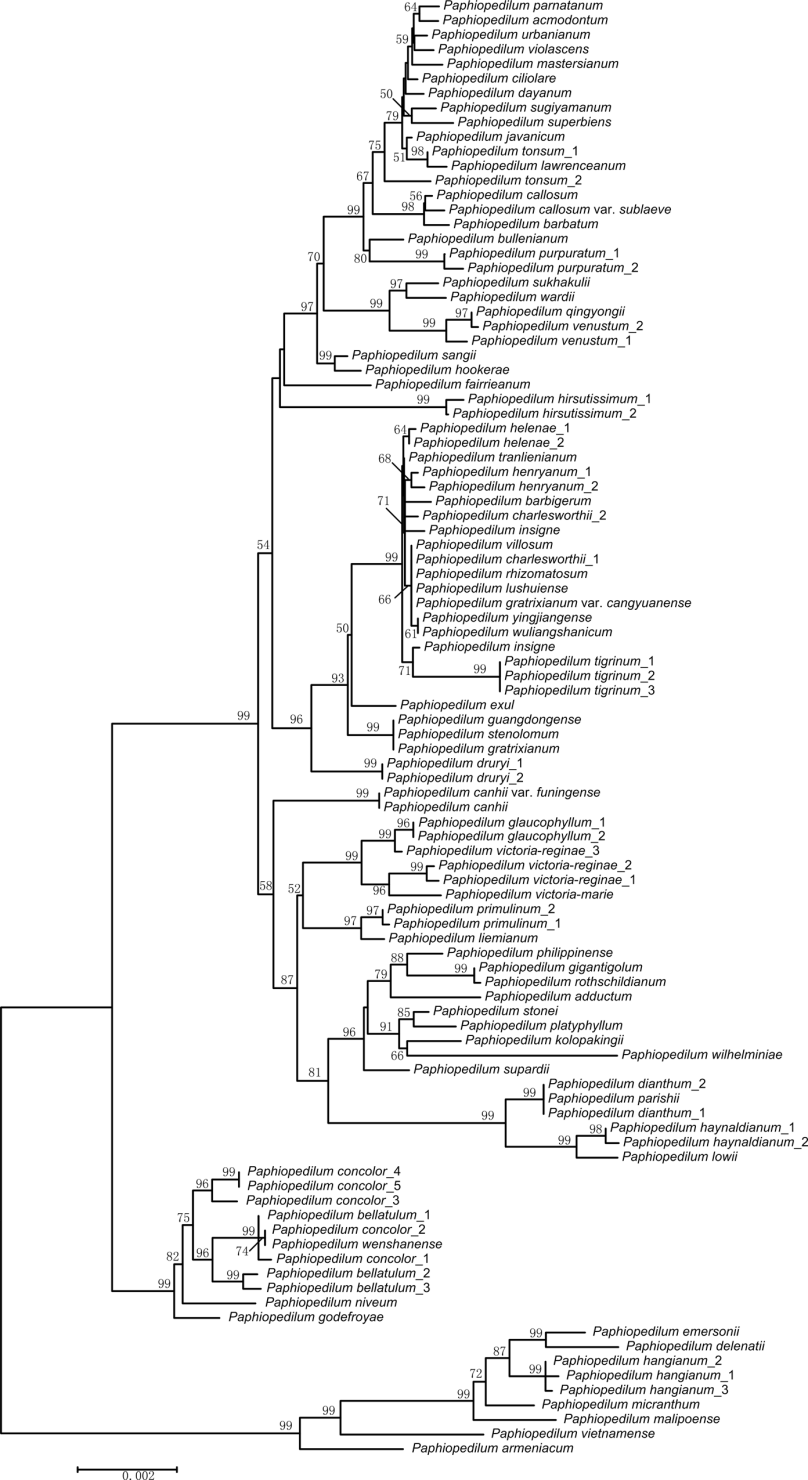
Neighbor-joining tree of *Paphiopedilum* based on the combination of the eight cpDNAs.

To eliminate the error induced by the sampling, we calculated the resolution of the taxa with the sequences downloaded from GenBank and the single-locus resolution increased significantly (Table 2), such that the resolution of *mat*K increased from 15.88% to 32.73% and the resolution of *ycf*1 increased from 22.42% to 31.13%. In addition, the accumulation curves for the haplotypes in the cpDNA and ITS indicated saturation of the candidate markers with the addition of the sequences from GenBank, which indicates the upper limit of the attendant barcodes (Fig 2). The tree topology of the NJ tree was congruent with that reported in previous studies [60,66]. However, several species represented by two or more individuals did not form monophyletic groups (Fig 3).

## Discussion

### The efficiency of the chloroplast markers in *Paphiopedilum*

Compared to the study of Parveen *et al*. [48], the identification rate decreased with denser species sampling (Table 2). The single-locus resolution ranged from 3.77% (*rbc*L) to 50.69% (ITS) (ITS > *rpo*C2 > *atp*F-*atp*H > *ycf*1 > *atp*I-*atp*H > *mat*K > *acc*D > *trn*S-*trn*fM > *rbc*L), but the single-locus can assign these species to Venus slipper. ITS is the most efficient single-locus barcode, identifying half the attendant sequences correctly and could be used easily as a potential barcode for Venus slipper. For the five coding cpDNA regions, the efficiency of *rbc*L is too low, whereas *ycf*1 and *rpo*C2 are too long to be used as barcodes (Table 1). The resolution of *mat*K is slightly higher than *acc*D and *mat*K is one of the most widely used phylogenetic markers with high variation. Therefore, we suggest *mat*K as one of the coding cpDNA regions for the identification of the Venus slipper, which is consistent with the results of Lahaye *et al*. [44] and Parveen *et al*. [48]. For the three intergenic regions, *atp*F-*atp*H has the highest resolution and shortest length compared to the other two regions (Tables 1, 2), and should be selected as a potential barcode. Moreover, the resolution of the combination of *mat*K and *atp*F-*atp*H is comparable with the other combinations.

For the other multi-locus combinations, the efficiency is similar, except for the two two-locus combination with relatively lower resolution (Table 2). The core plant barcode showed low efficiency in the Venus slipper (18.86%), which is much lower than the 72% obtained by the CBOL Plant Working Group [18] and this is not suitable to barcode the genus. In addition, the lengths of *mat*K, *atp*F-*atp*H, and ITS (Table 1) are also suitable as potential barcodes, which could be sequenced with one primer. Therefore, we recommend the combination of *mat*K + *atp*F-*atp*H + ITS as a barcode for Venus slipper during the preliminary stage.

### Factors that affect species discrimination

Fazekas *et al*. [67] demonstrated that the resolution of the plant dataset is ~70%. The resolution of the present study is relatively low compared to other orchid barcoding studies [44,46–48,50,51] and also non-orchid plant groups [21,25,35,37,38,40,41,67,68]. According to the evolution of the Venus slipper and the sampling strategy of this study, the factors that affect the species discrimination may include the recent diversification of many species, the upper limit of the barcodes, and/or the sampling density.

The common ancestor of the Venus slipper dates to the early Miocene [69] and many species are recently diverged [60]. Recently diverged species are difficult to identify [70]. For example, the successful identification of *Inga* species is 69% and 32% in *Araucaria* [68]. Most species of *Inga* originated from recent radiations [68]. In *Picea*, the recently diversified species distributed in the Himalayan–Hengduan Mountains and northeastern Asia are also a challenge for barcoding [25]. In young species, gene flow may blur the delimitation of closely related species. Guo *et al*. [60] determined that reticulate evolution plays an important role in the speciation of *Paphiopedilum* and the rampant non-monophyly of the tested species [43,60] (Fig 3) indicates that the Venus slippers are a conundrum for DNA barcoding.

The upper limit of the chloroplast genes also constrains the success rate of species identification [67]. In our study, the combination of the eight cpDNAs together did not significantly improve the resolution of this genus (Table 2), which indicates that the addition of other cpDNAs may lead to correct identification, but would not improve efficiency. In addition, the accumulation curves for the haplotypes in *mat*K, *ycf*1, and ITS show saturation, which suggests that the barcode efficiency reached the upper limit with increased sampling. There is no barcoding gap in the candidate barcodes of the genus (Fig 1). The barcoding gap does not exist in some other tested plant groups [22,25,44,71–73] and it also affects the upper limit of resolution in the Venus slipper and other untested plant groups. In Bromeliaceae, the two-locus (*mat*K + *rbc*L) species discrimination is 43.48% and the addition of a third locus (*trn*H-*psb*A) did not show a significant improvement [35].

The sampling density may also affect the efficiency. Our study covers 70–90% of the accepted species of Venus slipper. Parveen *et al*. [48] only sampled eight species of *Paphiopedilum*, which represent no more than 8% of the accepted species and those eight species are strongly diverged; therefore, *mat*K may identify the eight species correctly. In our study, the resolution of *mat*K is 32.73% and after the saturation of the haplotype, with additional sampling of this genus, the efficiency may decrease. With more multiple representation species included, the resolution may be much higher before the accumulation curve of the single-locus barcode reaches saturation, similar to the single-locus resolution of *mat*K, *rbc*L, and *ycf*1 increasing with the addition of sequences from GenBank (Table 2). Other studies showed high resolution with relatively small sampling. For example, Yao *et al*. [47] collected 17 species of *Dendrobium* and *Hologlossum* is a relatively small genus [46]. The rate of successful identification is low in species rich clades and several species-rich genera, such as *Pouteria*, *Inga*, *Eschweilera*, and *Ocotea*, showed little or no variation in cpDNA [74]. Furthermore, several studies with dense sampling showed low resolution [22,72,75]. For *Sisyrinchium*, the study sampled 185 accessions from 98 putative species and ITS only identified 30.61–38.78% of the species included [22], whereas Sun *et al*. [72] collected 148 accessions from 38 species and determined that *mat*K could discriminate only 23.26% of *Dioscorea* taxa.

## Conclusions

The potential application of DNA barcoding promotes the development and growth of the method. In this study, we selected eight chloroplast barcodes and ITS to evaluate their suitability in Venus slippers with dense sampling. We found that ITS is the most efficient single-locus barcode, which can identify half the Venus slippers correctly, whereas the combination of *mat*K + *atp*F-*atp*H is the most efficient multi-locus barcode. Therefore, we recommend the combination of *mat*K + *atp*F-*atp*H + ITS as the barcode for Venus slipper. However, there is an upper limit of the barcodes tested; therefore, adding more fragments apparently cannot solve the problem. Because of recent diversification and a complex evolutionary history in the genus, low-copy nuclear genes may be used in the DNA barcoding of this genus for more precise identification.

This study sheds light on the barcoding of orchids in a more efficient manner, which can improve orchid conservation. In the future, additional horticultural forms may be cultivated, which will lessen the over-collection from the natural environment. However, based on the assessment of the markers commonly used for the standardized application of this technique, much work remains to be done.

## Supporting Information

S1 Table Sources of materials. (DOC)
