# Supplementary material for: Promise and Challenge of DNA Barcoding in Venus Slipper (Paphiopedilum)
Source: PLoS One. 2016 Jan 11;11(1):e0146880. doi: 10.1371/journal.pone.0146880 (PMC4713476; doi:10.1371/journal.pone.0146880)
Supplement: S1 Table — (DOC) [file pone.0146880.s001.doc]

Supplementary Information

**Promise and challenge of DNA barcoding in Venus slipper (*Paphiopedilum*)**

Yan-Yan Guo ^a, b, c^, Lai-Qiang Huang ^b^, Zhong-Jian Liu ^a^, *, Xiao-Quan Wang ^c^, *

^a^ Shenzhen Key Laboratory for Orchid Conservation and Utilization, The National Orchid Conservation Center of China and The Orchid Conservation and Research Center of Shenzhen, Shenzhen 518114, China

^b^ Center for Biotechnology and BioMedicine, Graduate School at Shenzhen, Tsinghua University, Shenzhen 518055, China

^c^ State Key Laboratory of Systematic and Evolutionary Botany, Institute of Botany, Chinese Academy of Sciences, Beijing 100093, China

**Corresponding author:**

Zhong-Jian Liu: liuzj@sinicaorchid.org

Xiao-Quan Wang: xiaoq_wang@ibcas.ac.cn

S1 Table. Plant material and GenBank accession numbers.

S1 Table. Plant material and GenBank accession numbers.

| **Taxon name** | ***mat*K** | ***rbc*L** | ***ycf*1** | **ITS** |
| --- | --- | --- | --- | --- |
| **Subg. *Parvisepalum*** |  |  |  |  |
| Sect. *Parvisepalum* |  |  |  |  |
| *P. armeniacum* S.C.Chen & F.Y. Liu | EU490698  JQ660906 |  | EU490759 | Z78496  AY643431  EF156086  JQ660882  JX088560 |
| *P. delenatii* Guillaumin | AY368379  EU490699  JQ182193  JQ660905  JQ929368 | AF074208  JQ182211 | EU490760  JQ182265  JQ929521 | Z78497  EF156096  GU461600  JQ660881  JQ929314  JX088548 |
| *P. emersonii* Koop. & P.J.Cribb |  |  |  | Z78495  AJ564355  EF156099  GU993841  JX088544 |
| *P. hangianum* Perner & O.Gruss |  |  |  | EF156109  FJ712271  GU993843  JX088558 |
| *P*. *jackii* H.S.Hua |  |  |  | EF156118  HQ123427  HQ123428 |
| *P. malipoense* S.C.Chen & Z.H.Tsi | JQ929388 |  | JQ929541  KF361699 | Z78498  AJ564357  EF156125  FJ899755  GU993848  JQ929336  JX088554 |
| *P. micranthum* Tang & F.T.Wang | JQ929390 |  | JQ929543 | Z78499  AY643432  EF156127  EF156128  FJ712270  GU993849  JQ929338  JX088561 |
| *P. vietnamense* O.Gruss & Perner | JQ182194 | JQ182212 | JQ182266 | AY643433  EF156158  GU461603 |
| **Subg. *Brachypetalum*** |  |  |  |  |
| Sect. *Concoloria* |  |  |  |  |
| *P. bellatulum* (Rchb.f.) Stein | JN181448 | AF074207  JN181465 | JN181516 | Z78492  JX088553 |
| *P*. *concolor* (Lindl. ex Bateman) Pfitzer in H.G.A.Engler & K.A.E.Prantl (eds.) | JQ660902  JQ929367 |  | JQ929520 | Z78491  AJ564366  AY643435  EF156093  GU461599  JQ660878  JQ929312  JQ929313  JX088557 |
| *P. godefroyae* (God.-Leb.) Stein |  |  |  | Z78493  EF156106  EF156107 |
| *P*. *niveum* (Rchb.f.) Stein | JQ660903  JQ929391 |  | JQ929544 | AY643436  EF156130  JQ660879  JQ929339 |
| *P*. *wenshanense* Z.J. Liu & J.Yong Zhang |  |  |  | JQ904599 |
| **Subg. *Paphiopedilum*** |  |  |  |  |
| Sect. *Coryopedilum* |  |  |  |  |
| *P. adductum* Asher | JQ182191  JQ929361 | JQ182209 | JQ182263  JQ929514 | AY643459  EF156082  EF156083  JQ929305 |
| *P*. *coccineum* Perner & R.Herrm*.* |  |  |  | GU461598 |
| *P. gigantifolium* Braem |  |  |  | EF156103 |
| *P. glanduliferum* (Blume) Stein | JQ660887  JQ929373  JQ929374 |  | JQ929526  JQ929527 | Z78463  Z78464  AY643451  EF156104  JQ660864  JQ929319  JQ929320 |
| *P*. *intaniae* Cavestro |  |  |  | EF156117 |
| *P. kolopakingii* Fowlie | JQ929383 |  | JQ929536 | Z78474  AJ564359  EF156121  JQ929331 |
| *P*. *ooii* Koop. |  |  |  | EF156138 |
| *P. philippinense* (Rchb.f.) Stein | JQ929393 |  | JQ929546 | AY643475  EF156141  EF156142  JQ929341 |
| *P*. *praestans* (Rchb.f.) Pfitzer | JQ660886 |  |  | AY643452 |
| *P. randsii* Fowlie | JQ929396 |  | JQ929549 | AY643458  EF156132  JQ929344 |
| *P. rothschildianum* (Rchb.f.) Stein | JQ660888  JQ929397 |  | JQ929550 | Z78465  AJ564370  AY643453  EF156135  EF459731  JQ660865  JQ929345 |
| *P. sanderianum* (Rchb.f.) Stein |  |  |  | AJ564374  AJ564375  EF156136 |
| *P. stonei* (Hook.) Stein | JQ660889  JQ929400 |  | JQ929553 | Z78467  AJ564361  EF156146  JQ660866  JQ929348 |
| *P. supardii* Braem & Löb |  |  |  | AY643454  GQ505309 |
| *P. wilhelminae* L.O.Williams | JQ929408 |  | JQ929561 | AJ564371  AJ564373  AY643455  GQ505310  JQ929357 |
| Sect. *Pardalopetalum* |  |  |  |  |
| *P. dianthum* Tang & F.T.Wang | JQ182192  JQ929369 | JQ182210 | JQ182264  JQ929522 | Z78471  EF156097  GU461601  JQ929315  JX088562 |
| *P. haynaldianum* (Rchb.f.) Stein | JQ929379 | AB176547 | JQ929532 | AB176592  AF324175  EF156110  JQ929325 |
| *P. lowii* (Lindl.) Stein | JQ660890  JQ929386  JQ929387 |  | JQ929539  JQ929540 | Z78472  AY643456  EF156124  JQ660867  JQ929334  JQ929335 |
| *P. parishii* (Rchb.f.) Stein | JQ660891  JQ929392 |  | JQ929545 | EF156140  JQ660868  JQ929340  JX088555 |
| *P*. *richardianum* Asher & Beaman |  |  |  | AY643457  AY643474  EF156133 |
| Sect. *Cochlopetalum* |  |  |  |  |
| *P. glaucophyllum* J.J.Sm*.* | AY557205  JQ929375 |  | JQ929528 | AY643437  EF156105  JQ929321 |
| *P. liemianum* (Fowlie) K.Karas. & K.Saito | JQ929385 |  | JQ929538 | EF156123  JQ929333 |
| *P*. *moquetteanum* (J.J.Sm.) Fowlie |  |  |  | EF156129 |
| *P. primulinum* M. Wood & Taylor | JN181451  JQ929394 | JN181468 | JN181519  JQ929547 | Z78479  AY643438  EF156143  JQ929342 |
| *P*. *primulinum* var. *purpurascens* (M.W.Wood) P.J.Cribb | JQ929395 |  | JQ929548 | AY643439  JQ929343 |
| *P. victoria-mariae* (Sander ex Mast.) Rolfe |  |  |  | AY643440  EF156156 |
| *P. victoria-regina* (Sander) M.W.Wood | JQ660892  JQ660893  JQ929404 |  | JQ929557 | AJ564360  AY643441  EF156157  JQ660869  JQ660870  JQ929353 |
| Sect. *Paphiopedilum* |  |  |  |  |
| *P. barbigerum* Tang & F.T.Wang |  |  |  | Z78486  AY643442  EF156088  FJ899754  JX088559 |
| *P. charlesworthii* (Rolfe) Pfitzer | JQ929365 |  | JQ929518 | Z78484  EF156091  JQ929310  JX088552 |
| *P. druryi* (Bedd.) Stein | HQ998482  HQ998483  HQ998484  JQ660894  JQ929370 | HQ998557  HQ998558  HQ998559 | JQ929523 | Z78489  EF156098  HQ998448  HQ998449  HQ998450  JQ660871  JQ929316 |
| *P. exul* (Ridl.) Rolfe | JQ929371 |  | JQ92952 | GUEF156101  JQ929317 |
| *P. fairrieanum* (Lindl.) Stein | HQ998485  HQ998486  HQ998487  HQ998488  HQ998489 | HQ998560  HQ998561  HQ998562  HQ998563  HQ998564 |  | Z78490  AJ564367  AY643443  EF156102  HQ998451  HQ998452  HQ998453  HQ998454  HQ998455 |
| *P. fowliei* Birk | JQ929372 |  | JQ929525 | GQ505311  JQ929318 |
| *P. gratrixianum* Rolfe | JQ660900  JQ929376  JQ929377  JQ929378 |  | JQ929529  JQ929530  JQ929531 | AY643472  EF156108  FJ899753  GU993842  JQ660876  JQ929322  JQ929323  JQ929324  JX088549 |
| *P. helenae* Aver. | JQ660901 |  |  | EF156111  JQ660877  JX088550 |
| *P. henryanum* Braem |  |  |  | Z78485  AY643445  EF156112  GU993845  JX088551 |
| *P. hirsutissimum* (Lindl. Ex Hook.) Stein | HQ998490  HQ998491  HQ998492  HQ998493  HQ998494  JN181449 | HQ998565  HQ998566  HQ998567  HQ998568  HQ998569  HQ998570  JN181466 | JN181517  KF361698 | Z78487  AJ564368  AY643446  AY643447  AY643473  EF156100  EF156114  GU993847  HQ998456  HQ998457  HQ998458  HQ998459  HQ998460  HQ998461  JQ929327  JQ929328  JX088547 |
| *P. insigne* (Wall. ex Lindl.) Pfitzer | HQ998496  HQ998497  JQ660898  JQ929381 | HQ998571  HQ998572  HQ998573  HQ998574 | JQ929534 | AJ564369  AY643448  AY643449  HQ998462  HQ998463  HQ998465  HQ998466  JQ660874  JQ929329 |
| *P. rhizomatosum* S.C.Chen & Z.J.Liu |  |  |  | JQ904598 |
| *P. spicerianum* (Rchb.f.) Pfitzer | HQ998504  HQ998505  HQ998506  HQ998507  JQ929399 | HQ998580  HQ998581  HQ998582  HQ998583  HQ998584 | JQ929552 | AY643450  EF156145  HQ998467  HQ998468  HQ998469  HQ998470  JQ929347  JX088545 |
| *P. tigrinum* Koop. & N.Haseg |  |  |  | Z78488  EF156149  JQ929351 |
| *P. tranlienianum* O.Gruss & Perner |  |  |  | EF156151  FJ712267  GU461602  JX088556 |
| *P. villosum* (Lindl.) Stein | JQ660899  HQ998514 | HQ998592  HQ998593  HQ998594  HQ998595  HQ998596 |  | Z78483  EF156159  FJ712268  FJ712269  GU993851  JQ660875  JX088563  HQ998476  HQ998477  HQ998478  HQ998479  HQ998480 |
| *P. villosum* var. *boxallii* (Rchb.f.) Pfitzer in H.G.A.Engler (ed.) | HQ998515  JQ929405 |  | JQ929558 | JQ929354 |
| Sect. *Barbata* |  |  |  |  |
| *P. acmodontum* M.W.Wood |  |  |  | Z78446  EF156081 |
| *P. appletonianum* (Gower) Rolfe | JQ929362 |  | JQ929515 | EF156084  GU461596  JQ929306 |
| *P. argus* (Rchb.f.) Stein | JQ660896 |  |  | Z78448  AJ564363  EF156085  JQ660873 |
| *P. barbatum* (Lindl.) Pfitzer | JQ660895  JQ929363 |  | JQ929516 | EF156087  JQ660872  JQ929307 |
| *P*. *bougainvilleanum* Fowlie |  |  |  | Z78452 |
| *P*. *braemii* H.Mohr |  |  |  | AY643463  AY643464  EF156089 |
| *P. bullenianum* (Rchb.f.) Pfitzer |  |  |  | Z78442  AY643465  EF156134 |
| *P. callosum* (Rchb.f.) Stein | JQ929364 |  | JQ929517 | Z78457  AJ564365  EF156090  GU461597  JQ929308 |
| *P*. *callosum* var. *sublaeve* (Rchb.f.) P.J.Cribb |  |  |  | JQ929309 |
| *P. ciliolare* (Rchb.f.) Stein | GU120221  JQ929366 |  | JQ929519 | Z78460  EF156092  GU120211  JQ929311 |
| *P. dayanum* (Lindl.) Stein | GU120219 |  |  | Z78459  EF156095  GU120209 |
| *P. hennisianum* (M.W.Wood) Fowlie | JQ929380 |  | JQ929533 | Z78458  AY643460  JQ929326 |
| *P. hookerae* (Rchb.f.) Stein |  |  |  | Z78451  EF156116 |
| *P*. *hookerae* var. *volonteanum* (Sander ex Rolfe) Braem |  |  |  | EF156115 |
| *P. javanicum* (Reinw. ex Lindl.) Pfitzer | GU120220  GU120222  JQ929382 |  | JQ929535 | Z78455  AY643461  EF156119  EF156120  GU120210  GU120212  JQ929330 |
| *P. lawrenceanum* (Rchb.f.) Pfitzer | JQ929384 |  | JQ929537 | EF156122  JQ929332 |
| *P. mastersianum* (Rchb.f.) Stein | GU120217  JQ929389 |  | JQ929542 | Z78449  AY643466  EF156126  GU120207  JQ929337 |
| *P. mastersianum* var. *mohrianum* (Braem) Koop. | GU120218 |  |  | GU120208 |
| *P*. *papuanum* (Ridl. ex Rendle) L.O.Williams |  |  |  | Z78450 |
| *P. purpuratum* (Lindl.) Stein |  |  |  | Z78440  AJ564364  EF156131  GU993850  JX088564 |
| *P. sangii* Braem | JQ929398 |  | JQ929551 | EF156137  JQ929346 |
| *P. schoseri* Braem & H.Mohr | GU120214 |  |  | Z78453  AY643462  EF156144  GU120204 |
| *P. sugiyamanum* Cavestro | GU120215 |  |  | GU120205 |
| *P. sukhakulii* Schoser & Senghas | JQ929401 | AF074209 | JQ929554 | AJ564362  AY643468  EF156147  JQ929349 |
| *P. superbiens* (Rchb.f.) Stein | JQ660897  JQ929402 |  | JQ929555 | Z78441  AY643467  EF156094  EF156148  JQ929350 |
| *P. tonsum* (Rchb.f.) Stein | GU120216  JQ929403 |  | JQ929556 | Z78456  EF156150  GU120206  JQ929352 |
| *P. urbanianum* Fowlie |  |  |  | Z78445  EF156152 |
| *P. venustum* (Wall. ex Sims) Pfitzer | HQ998510  HQ998511  HQ998512  HQ998513 | HQ998587  HQ998588  HQ998589  HQ998590  HQ998591 |  | Z78447  EF156154  EF156155  HQ998471  HQ998472  HQ998473  HQ998474  HQ998475 |
| *P*. *viniferum* Koop. & N.Haseg. |  |  |  | EF156153 |
| *P. violascens* Schltr. | JQ929406 |  | JQ929559 | EF156160  JQ929355 |
| *P. wardii* Summerh. | HQ998516  JN181450  JQ929407 | HQ998597  JN181467 | JN181518  JQ929560 | AY643469  EF156161  HQ998481  JQ929356  JX088546 |
| Subg. *Megastaminodium* |  |  |  |  |
| *Paphiopedilum canhii Aver. & O.Gruss* | JQ660904 |  |  | JQ660880 |
| *Paphiopedilum canhii* var. *funingense* Z.J.Liu & L.J.Chen |  |  |  |  |
| Hybrid |  |  |  |  |
| *P*. × *herrmannii* F.Fuchs & H.Reisinger |  |  |  | EF156113 |
| *P.* × *lushuiense* Z.J.Liu & S.C.Chen |  |  |  |  |
| *P*. × *pradhanii* Pradhan | HQ998500  HQ998501  HQ998502  HQ998503 | HQ998575  HQ998576  HQ998577  HQ998578  HQ998579 |  |  |
| *P.* × *venustoinsigne* Pradhan | HQ998508 | HQ998585 |  |  |
